# Quantitative Proteomics of Human Retinal Pigment Epithelium Reveals Key Regulators for the Pathogenesis of Age-Related Macular Degeneration

**DOI:** 10.3390/ijms24043252

**Published:** 2023-02-07

**Authors:** Shichen Shen, Rebecca J. Kapphahn, Ming Zhang, Shuo Qian, Sandra R. Montezuma, Peng Shang, Deborah A. Ferrington, Jun Qu

**Affiliations:** 1 Department of Pharmaceutical Sciences, University at Buffalo, Buffalo, NY 14214, USA; 2 New York State Center of Excellence in Bioinformatics and Life Sciences, Buffalo, NY 14203, USA; 3 Department of Ophthalmology and Visual Neurosciences, University of Minnesota, Minneapolis, MN 55455, USA; 4 Department of Cell Stress Biology, Roswell Park Comprehensive Cancer Center, Buffalo, NY 14203, USA; 5 Doheny Eye Institute, Pasadena, CA 91103, USA

**Keywords:** age-related macular degeneration, retinal pigment epithelium, mitochondria dysfunction, quantitative proteomics, mass spectrometry, IonStar

## Abstract

Age-related macular degeneration (AMD) is the leading cause of blindness in elderly people, with limited treatment options available for most patients. AMD involves the death of retinal pigment epithelium (RPE) and photoreceptor cells, with mitochondria dysfunction being a critical early event. In the current study, we utilized our unique resource of human donor RPE graded for AMD presence and severity to investigate proteome-wide dysregulation involved in early AMD. Organelle-enriched fractions of RPE were isolated from donors with early AMD (*n* = 45) and healthy age-matched controls (*n* = 32) and were analyzed by UHR-IonStar, an integrated proteomics platform enabling reliable and in-depth proteomic quantification in large cohorts. A total of 5941 proteins were quantified with excellent analytical reproducibility, and with further informatics analysis, many biological functions and pathways were found to be significantly dysregulated in donor RPE samples with early AMD. Several of these directly pinpointed changes in mitochondrial functions, e.g., translation, ATP metabolic process, lipid homeostasis, and oxidative stress. These novel findings highlighted the value of our proteomics investigation by allowing a better understanding of the molecular mechanisms underlying early AMD onset and facilitating both treatment development and biomarker discovery.

## 1. Introduction

Age-related macular degeneration (AMD) accounts for the majority of progressive and irreversible vision loss in the elderly population, affecting ~30% of individuals over 75 years old in developed countries [1]. A previous study estimated that ~290 million individuals will suffer from AMD by the year 2040, which imposes heavy burdens on human health worldwide [2]. AMD is characterized by macular deterioration, resulting in central vision loss and subsequent difficulties in accomplishing daily activities that require visualization of fine details, such as reading and face recognition. Most patients manifest a “dry” clinical phenotype that involves loss of retinal pigment epithelium (RPE) and death of photoreceptors [3]. Treatment options for dry AMD include a nutritional supplement that has been shown to provide moderate benefits for a limited proportion of dry AMD patients. However, exacerbation of AMD was still observed in most of the participating patients [4,5]. Thus, more effective therapeutic strategies directly targeting the underlying molecular mechanisms of dry AMD are highly favorable.

Mitochondria are vital organelles responsible for a variety of cellular functions, including energy production, regulation of cell survival and death, control of redox signaling, and cation homeostasis [6]. As the primary energy source in the RPE, mitochondria are essential for normal RPE functions and hence compromised mitochondrial function is directly associated with RPE dysfunction and cell death. Recent studies have found that cultured RPE cells from human donors with AMD manifested declined oxidative phosphorylation compared with those from non-AMD donors, suggesting that mitochondria dysfunction may be one of the underlying mechanisms driving AMD pathology [7]. Moreover, we have also demonstrated that primary cultures of RPE from AMD donors showed AMD-dependent responses to multiple mitochondria-targeting drugs [8]. These positive outcomes were not observed in cultured RPE from non-AMD human donors, indicating the potential of mitochondria-targeting drugs as an option for AMD treatment.

To elucidate the molecular basis underlying AMD pathology, especially during disease onset, a more in-depth understanding of why mitochondria dysfunction occurs in AMD is required. We propose that the proteomic profile associated with the transition from healthy aging to early AMD will provide key insights into the mechanism driving AMD pathology. In this study, we utilized a proteomic approach to characterize changes in RPE protein content in AMD donors compared with age-matched non-AMD controls. To better quantify organelle proteins, the majority of nuclear, cytosolic, and membrane proteins were removed from the whole RPE lysates via differential centrifugation. This commonly used method of enriching cytoplasmic organelles reduces sample complexity, thereby improving the ability of distinguishing changes in protein content. In our previous studies, proteomic investigations of human RPE at progressive stages of AMD revealed dysregulation of key signaling pathways implying mitochondria dysfunction, including mitochondrial trafficking, translation, apoptosis, and ATP synthase activity [9]. Of note, these investigations were accomplished using two-dimensional (2-D) gel electrophoresis coupled to matrix-assisted laser desorption ionization time-of-flight (MALDI-TOF) mass spectrometry (MS). While this technology has been ubiquitously utilized for several decades, it has become gradually outdated due to limited sensitivity, reproducibility, and depth, especially when quantitative analysis of the proteome is warranted. With the introduction of nano-flow liquid chromatography (LC) and electrospray ionization (ESI), as well as the advent of ultra-high-field orbitrap mass spectrometers [10], more comprehensive and unbiased proteomics analysis with deep proteome coverage and excellent quality for quantification is now achievable. Thus, the current study significantly extends the capabilities of our previous analysis to identify altered proteins in human RPE samples.

**Figure 1:**
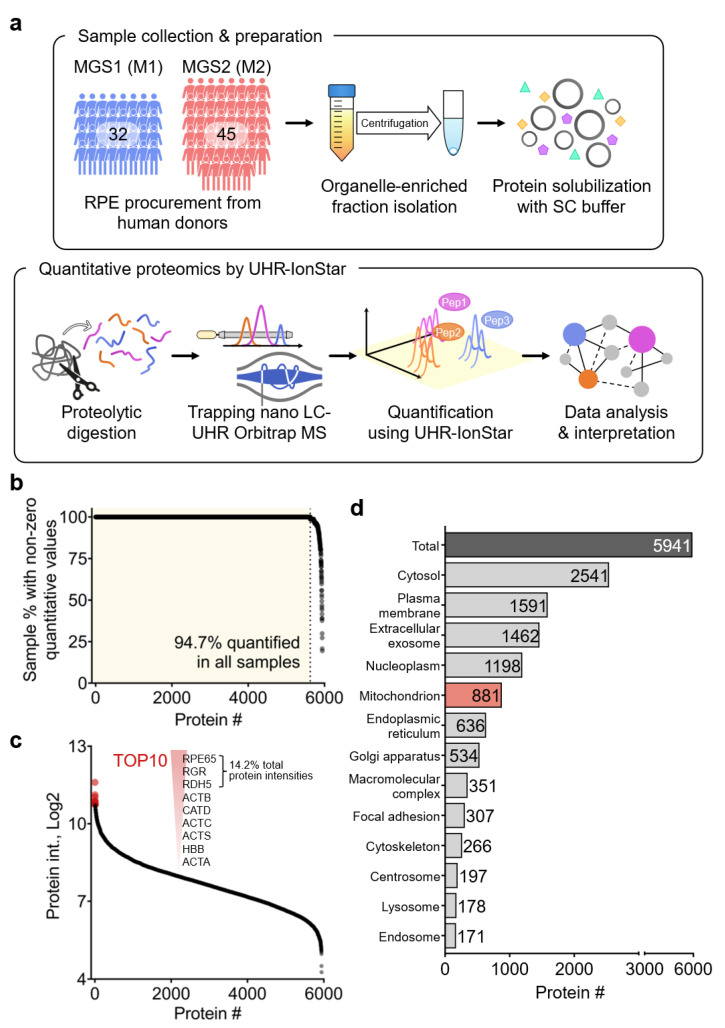
Proteomics profiling of RPE organelle-enriched fractions from human donors with AMD. (**a**) Experimental and data analysis procedures of the current study; (**b**) the percentage of proteins quantified with non-zero quantitative values (i.e., missing data-free) in the dataset; (**c**) ranked order of protein MS intensities and the TOP10 most abundant proteins. (**d**) Gene ontology (GO) enrichment of cellular component (CC) terms for the 5941 proteins quantified.

Here we present a quantitative proteomics investigation of organelle-enriched fractions of RPE from human donors with early-stage AMD and age-matched healthy controls. The presence and severity of AMD were determined using the Minnesota Grading System (MGS), with donors exhibiting no evidence of AMD designated as MGS1 and donors with early AMD as MGS2 [11]. It is important to note that donors with early AMD (MGS2) have experienced no vision loss and, upon ophthalmic exam, exhibit no RPE or photoreceptor loss. The only clinical evidence of disease is the presence of a few small deposits, known as drusen, in the retina. Organelle-enriched fractions were isolated from individual RPE samples (*n* = 77 in total) and were analyzed using UHR-IonStar, a unique label-free quantitative proteomics platform devised for highly reproducible and reliable proteomics analysis in large sample cohorts (Figure 1a) [12,13]. Isolated fractions were first processed by a surfactant cocktail (SC)-aided precipitation/on-pellet digestion (SEPOD) protocol for protein solubilization and digestion [14] and were then analyzed by a large-capacity trapping nano-flow LC-ultra-high-resolution (UHR) orbitrap MS procedure [15]. Protein quantification was performed by the UHR-IonStar data processing pipeline, which allowed reliable proteomic quantification in large cohorts with high accuracy/precision and an extremely low false biomarker discovery rate [13]. Quantification results were further interpreted by a series of informatics approaches to identify proteins and corresponding biological functions/signaling pathways dysregulated in donors with early AMD.

## 2. Results

### 2.1. Quantification of RPE Proteome with UHR-IonStar

In the current study, a large cohort quantitative proteomics experiment was performed to investigate proteome-wide expression changes in RPE organelle-enriched fractions from human donors with early-stage AMD (MGS2/M2, *n* = 45) and without AMD (MGS1/M1, *n* = 32). Donors were matched in age and gender (Table S1). The average age of donors was 76.8 (±10.6) for MGS1 and 79.8 (±10.4) for MGS2. There was no significant difference in donor age between MGS2 and MGS1 (*p* = 0.22), and equal distribution of females and males was present in each group (MGS1 = 16:16 and MGS2 = 22:23). Under the identification criteria of 1% protein/peptide false discovery rate (FDR) and ≥2 unique peptides per protein, a total of 5941 unique proteins were quantified from the 77 samples (Table S2). An average of 5910 proteins were quantified in each sample (Figure S1a), and 94.7% of proteins were deemed as “missing data-free” (i.e., having a non-zero quantitative value in all 77 samples) in the entire sample set (Figure 1b). MS intensities of proteins quantified spanned 7.3 orders of magnitude, and the TOP3 most abundant proteins quantified were retinoid isomerohydrolase (RPE65), retinal G protein-coupled receptor (RGR), and retinol dehydrogenase (RDH5), which were all RPE-specific proteins and covered 14.2% of total protein MS intensities (Figure 1c). Gene ontology (GO) analysis of cellular component terms enriched a number of major subcellular compartments (Figure 1d), including cytosol (2541), plasma membrane (1591), extracellular exosome (1462), nucleoplasm (1198), mitochondrion (881), Endoplasmic reticulum (636), Golgi apparatus (534), cytoskeleton (266), centrosome (197), lysosome (178), and endosome (171). The median MS intensities of several subcellular organelles were much higher than those of all proteins quantified in the dataset, including mitochondria (2.4-fold higher; Figure S2). Endoplasmic reticulum (ER) was also enriched in the organelle fraction, which was expected considering the close association between ER and mitochondria, allowing their co-isolation with the centrifugation step. These results suggest that the sample preparation procedures achieved the anticipated outcomes with good robustness.

**Figure 2:**
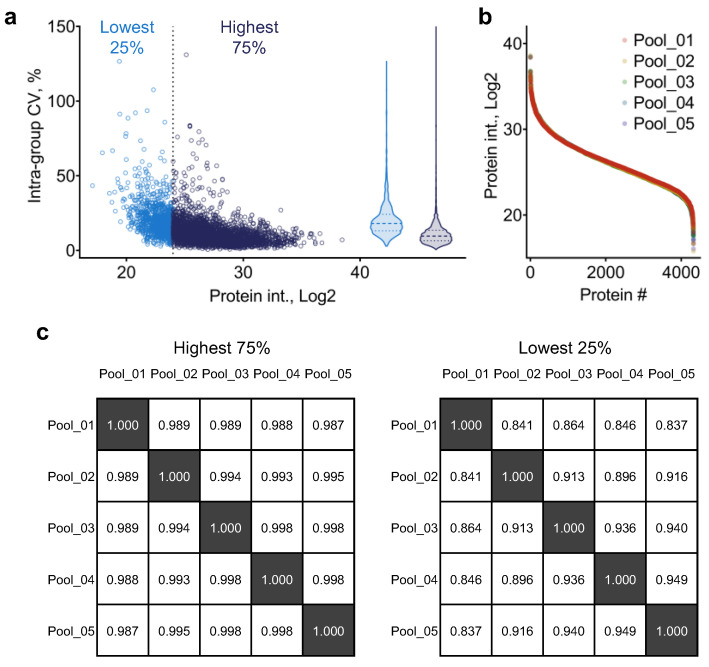
Assessment of LC-MS reproducibility during the analysis queue using the quality control (QC) sample. (**a**) Protein MS intensity-intra-group coefficient of variance (CV) plot. Proteins in the highest 75% and lowest 25% abundance range were shown in dark and light blue color; (**b**) ranked order of protein MS intensities for the 5 QC sample runs; (**c**) Pearson correlation matrices of the QC sample runs using proteins in the highest 75% and lowest 25% abundance range.

Considering the substantial size of the sample cohort, stringent quality control of the experimental procedures is crucial to the analytical quality of the quantitative results. We devised and applied a standardized scheme for the monitoring and evaluation of LC-MS reproducibility during the analysis queue (Figure S3). In brief, a quality control (QC) sample consisting of pooled aliquots of 10 randomly selected samples from the cohort being analyzed was injected for analysis at a fixed interval during LC-MS analysis. In this study, the QC sample was analyzed every 20 LC-MS runs (*n* = 5 in total), and these QC sample files were also included with the real sample files in the data processing pipeline and employed as the reference for chromatographic alignment (i.e., retention time adjustment and peak clustering). Quantification results of the five QC runs are included in Table S3. The median CV among the five QC runs was 9.6 ± 8.9% and 18.0 ± 12.2% for proteins within the highest 75% and lowest 25% ranges, respectively. The scatterplot (Figure 2a) shows that under most circumstances, the CV levels of individual proteins were negatively correlated with protein MS intensities. These results were consistent with our previous results assessing the quantitative reproducibility of UHR-IonStar. Additionally, protein intensity distribution was almost identical among the five QC sample runs, and an excellent correlation coefficient was achieved for proteins within the highest 75% and lowest 25% range (Figure 2b,c). Together, these results suggest that LC-MS reproducibility was well maintained during the analysis of the current sample cohort, laying a solid foundation for the subsequent analytical procedures.

### 2.2. Evaluation of Blood Protein Contamination for RPE Sample Qualification

**Figure 3:**
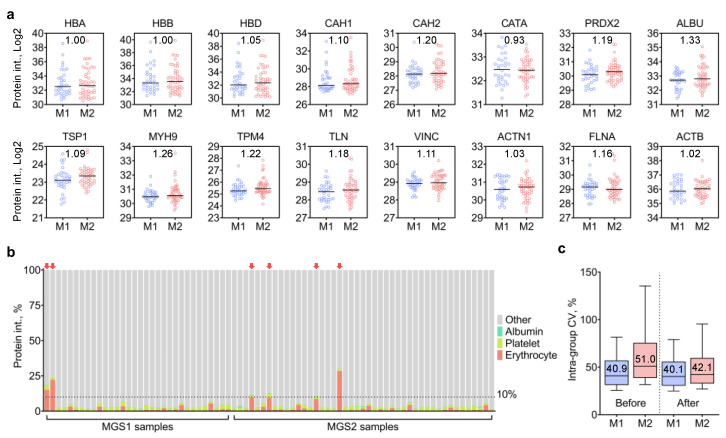
RPE sample qualification by evaluating blood protein contamination. (**a**) Protein MS intensities of the 16 selected blood proteins (7 erythrocyte-specific proteins, 8 platelet-specific proteins, 1 serum albumin) in MGS1 (M1) and MGS2 (M2) samples; (**b**) percentage of blood proteins (protein MS intensities) in individual samples; arrows indicate samples above the 10% threshold; (**c**) intra-group CV levels for MGS1 and MGS2 groups before and after removal of outlier samples.

RPE samples were removed from the human donor eye via manual harvesting using a fine tool. This process could potentially disrupt the blood-retina barrier and allow blood from the underlying choroid to be harvested along with the cells. AMD donors could be more susceptible to disruption of the blood-retina barrier since pathological changes in Bruch’s membrane, which separates the RPE from the outer retina blood supply of the choroid, have been well described [16]. Therefore, blood contamination in RPE samples from human donors is an important factor to consider since this could affect proteomics analysis, especially for relative quantification where high content of blood proteins could severely distort the overall data distribution. The most prominent sources of blood proteins are erythrocytes and platelets, while serum albumin is another major source of blood protein contamination. One previous study by Geyer et al. described a panel of quality-associated protein markers for plasma sample qualification, which encompasses numerous erythrocyte- and platelet-specific proteins that may also be applicable for the qualification of RPE samples from human donors [17]. Hence, we assessed the extent and impacts of blood protein contamination in the current sample set by compiling a list of sample quality markers based on the guidelines from the Geyer study. These included seven erythrocyte-specific proteins (HBA, HBB, HBD, CAH1, CAH2, CATA, PRDX2), eight platelet-specific proteins (TSP1, MYH9, TPM4, TLN, VINC, ACTN1, FLNA, ACTB), and serum albumin (ALBU). Assessment results are shown in Figure 3.

To begin, we calculated the ratios between MGS2 and MGS1 for the 16 sample quality markers selected to examine whether blood protein contamination was more severe in human donors with AMD. Among the sixteen quality markers selected, three erythrocyte-specific proteins (CAH, CAH2 and PRDX2), five platelet-specific proteins (VINC, FLNA, MYH9, TPM4, TLN1) and ALBU were increased by at least 10% in the MGS2 group (Figure 3a), confirming the increased likelihood of blood contamination in AMD samples. As shown in Figure 3b and Table S4, the average percentage of the 16 quality markers within each sample was 4.31 ± 1.06% (erythrocyte: 2.32 ± 1.96%; platelet: 1.79 ± 0.32%; albumin: 0.20 ± 0.63%). Interestingly, no correlation was observed between the percentage of erythrocyte- and platelet-specific proteins in individual samples (Figure S4). Based on the results, four MGS2 samples and two MGS1 samples with an excessive amount of blood proteins (i.e., >10% total MS intensities), which were considered as outlier samples, were excluded from the dataset (Figure 3b, highlighted by red arrows), along with one additional sample manifesting aberrantly high protein intensities (Figure S1b). The removal of outlier samples rendered the intra-group CV levels more similar between the two groups (40.1% and 42.1% for MGS1 and MGS2, respectively) and significantly decreased the intra-group CV levels from 51.0% to 42.1% for the MGS2 group. Quantification results after the removal of outlier samples (*n* = 70) can be found in Table S5.

### 2.3. Identification of Proteomic Dysregulation in MGS1 and MGS2 Donor Samples

**Figure 4:**
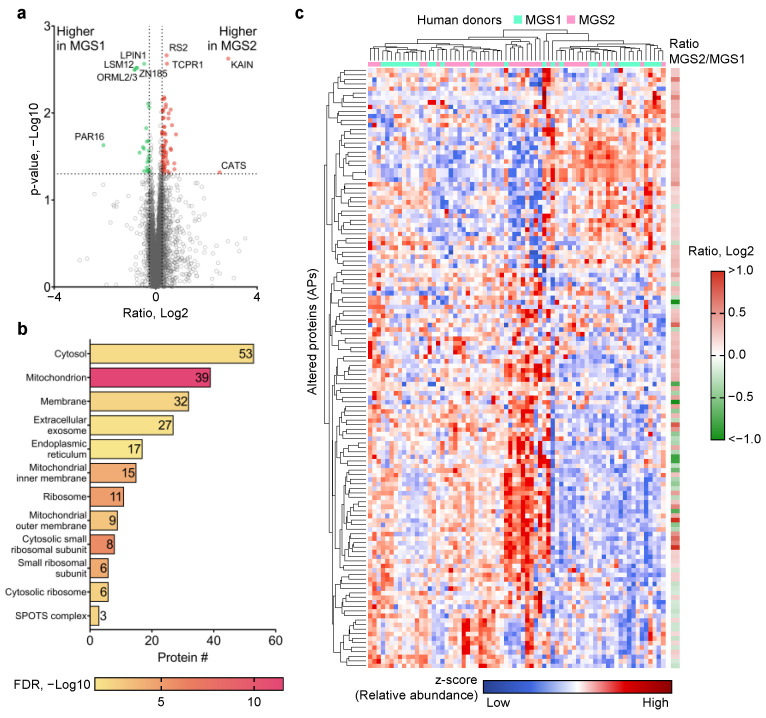
Determination of RPE proteome-wide changes between early AMD donors (i.e., MGS2) and healthy controls (i.e., MGS1). (**a**) Volcano plot of protein ratios and p-values. A total of 132 proteins were statistically significant, among which 67 and 26 were up- and downregulated; (**b**) GO enrichment of cellular components terms for the altered proteins (APs). A total of 39 APs were enriched in the mitochondria term with high statistical significance; and (**c**) heatmap showing results from unsupervised hierarchical clustering of the 132 APs in individual samples.

In order to identify proteome-wide dysregulation of protein expression in the organelle-enriched fraction of RPE samples, Welch’s test was performed between the MGS1 (*n* = 30) and MGS2 (*n* = 40) groups after outlier sample removal. A total of 132 proteins (Table S6), termed altered proteins (APs), were determined as statistically significant (i.e., *p*-value < 0.05). Principal component analysis (PCA) using the APs showed moderate though incomplete segregation of the MGS1 and MGS2 samples (Figure S5), suggesting that the RPE proteomic profiles reflect subtle shifts in discrete portions of the proteome in early disease. Among these APs, 67 and 26 proteins were up- and downregulated by at least 20% (i.e., Log_2_ MGS2/MGS1 ratio >0.25 or <−0.25; Figure 4a and Figure S5) in the MGS2 group. The top5 upregulated APs with the largest fold change were KAIN, CATS, BT3A3, TPP2, and GRAA. The top5 downregulated APs with the largest fold change were PAR16, ORML3, ORML2, LSM12, and ZN185 (Figure S6). GO analysis of Cellular Components led to the enrichment of 39 mitochondrion proteins with high statistical significance (−Log_10_FDR = 11.6; (Figure 4b). The 39 mitochondrial proteins are disproportionately represented (30%) among the APs when considering that mitochondrial proteins were only 15% of the total proteins identified (Figure 1). These data support the idea that mitochondrial protein expression is dysregulated in early AMD, which is consistent with other studies that suggest mitochondria dysfunction is a key event of early AMD pathogenesis. Additionally, APs were also enriched in several other key subcellular compartments, including cytosol (53), membrane (32), extracellular exosome (27), endoplasmic reticulum (17), and ribosome (11). Unsupervised hierarchical clustering of both APs and samples enabled the clustering of most MGS2 samples in the middle of the heatmap, while most of the MGS1 samples were separately localized on the two horizontal ends of the heatmap (Figure 4c). These results suggest that RPE at early-stage AMD may exhibit a signature pattern of protein dysregulation, which could contribute as etiological factors for AMD pathogenesis and has the potential to be established as protein markers for AMD onset.

**Figure 5:**
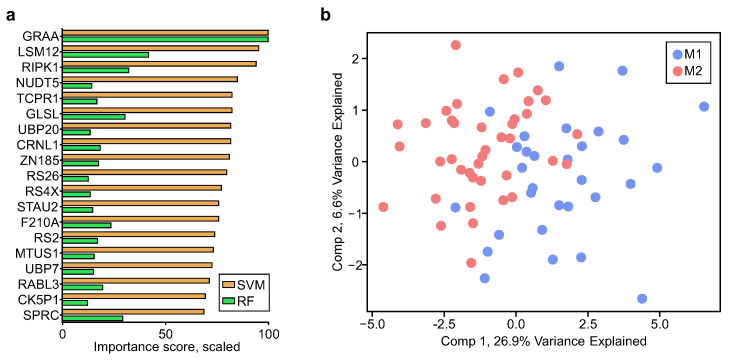
Supervised machine learning analysis of the 132 APs using random forest (RF) and support vector machine (SVM) algorithms. (**a**) Scaled importance of the 19 proteins overlapped between the TOP30 proteins with the highest importance in both models; (**b**) partial least squares-discriminant analysis (PLS-DA) results of the 19 proteins.

To evaluate this hypothesis, we employed two supervised machine learning methods, random forest (RF) and support vector machine (SVM), to develop predictive models distinguishing donors from MGS2 and MGS1 groups. Based on tenfold cross-validation (repeated three times) using the Log2 protein intensities of the 132 APs, RF and SVM achieved a similar accuracy of 0.710 (95% CI [0.639, 0.780]) and 0.738 (95% CI [0.686, 0.790]), respectively. We further trimmed down the list of APs by calculating feature importance metrics in RF and SVM models to identify APs with the highest contribution to classifiers. A total of 19 proteins overlapped among the TOP30 proteins, with the highest importance in both models (Figure 5a and Table S7). Partial least squares-discriminant analysis (PLS-DA) of these 19 proteins showed the segregation of most samples in MGS2 and MGS1 groups. Considering that MGS2 donors manifested minimal pathological changes in RPE and the relatively high intra-group CV levels of clinical samples, these results, to some extent, validate our hypothesis and indicate that proteome-level dysregulation may precede most macroscopic changes in AMD pathology.

**Figure 6:**
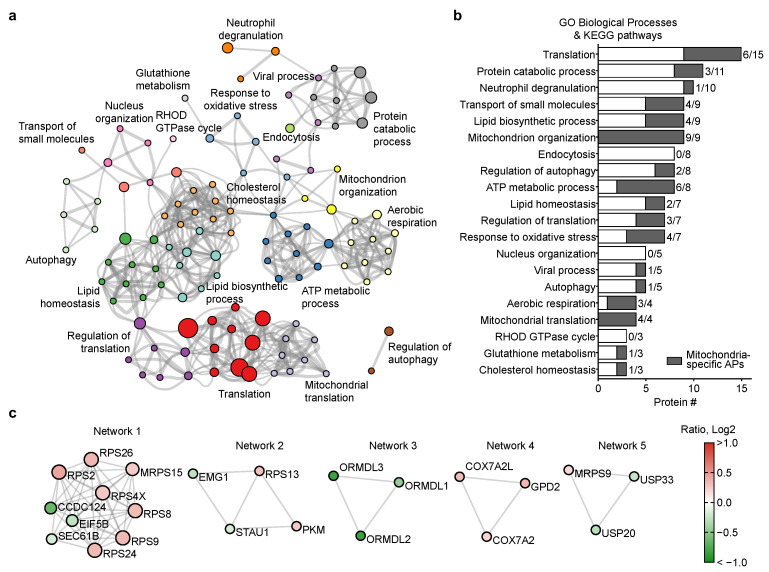
Functional enrichment of APs. (**a**) Clusters of biological functions and signaling pathways enriched. Protein number in each biological function/signaling pathway is proportional to the size of the node. (**b**) The number of APs in each cluster enriched. Mitochondria-localized APs are shown in gray. (**c**) Protein-protein interaction (PPI) networks enriched.

A functional analysis of the APs was then performed using Metascape. A total of 20 clusters of biological functions and signaling pathways were enriched from the 132 APs identified (Figure 6a), and based on functional proximity, these clusters can be classified into several categories: (i) translation-related: translation (15), regulation of translation (7), mitochondrial translation (4); (ii) energy metabolism-related: ATP metabolic process (8), aerobic respiration (4); (iii) lipid metabolism-related: lipid biosynthetic process (9), lipid homeostasis (7), cholesterol homeostasis (3); (iv) oxidative stress-related: response to oxidative stress (7), glutathione metabolism (3); (v) innate immunity-related: neutrophil degranulation (10), viral process (5); (vi) miscellaneous: protein catabolic process (11), transport of small molecules (9), mitochondrion organization (9), endocytosis (8), regulation of autophagy (8), nucleus organization (5), autophagy (5), and RHOD GTPase cycle (3). We then mapped the 39 mitochondria-localized APs to each of these clusters and found that mitochondria-localized APs were present in 17 out of the 20 clusters, implicating that mitochondrion, potentially in a dysregulated state, was contributing to early AMD pathology (Figure 6b). Moreover, 5 out of the 20 clusters consisted of >50% mitochondria-localized APs, including mitochondrial translation (4/4, 100%), mitochondrion organization (9/9, 100%), ATP metabolic process (6/8, 75%), aerobic respiration (3/4, 75%), and response to oxidative stress (4/7, 57.1%). Furthermore, five protein–protein interaction (PPI) networks were established. Among these PPI networks, Network 1 consists of seven ribosomal proteins, including one mitochondrial ribosomal protein (MRPS15); Network 3 consists of three ORM1-like proteins involved in sphingolipid metabolism; Network 4 encompasses three mitochondria-localized proteins involved in aerobic respiration (GPD2, COX7A2L, COX7A2).

**Figure 7:**
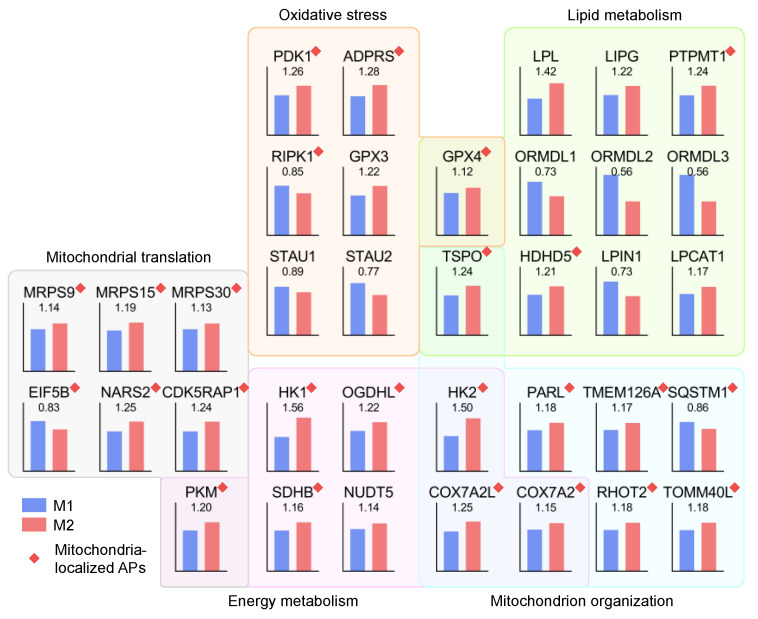
Normalized intensities and inter-group ratios of 36 APs in the five selected functional categories.

To further explore the relationships between the altered protein levels in the current dataset and mitochondria dysfunction as a putative contributor for AMD onset, we selected five specific functional categories with high percentages of mitochondria-localized APs based on their biological roles in maintaining normal mitochondrial functions. These categories include mitochondrial translation (7), oxidative stress (7), lipid metabolism (11), energy metabolism (8), and mitochondrion organization (9). As shown in Figure 7, 25 out of the 36 APs selected were localized in mitochondria, and 27/9 proteins were significantly up- or downregulated in the MGS2 group. Of the three categories directly related to mitochondria functions (i.e., mitochondrial translation, energy metabolism, and mitochondrion organization), the majority of proteins were upregulated (18 out of 20). Interestingly, all seven APs involved in energy metabolism were upregulated in the MGS2 group, including three proteins in glycolysis (HK1, HK2, PKM), two proteins in the Krebs cycle (SDHB, OGDHL), two proteins in oxidative phosphorylation (COX7A2L, COX7A2), and one non-mitochondria-localized protein (NUDT5). Moreover, five out of six mitochondrial proteins involved in translation, including three ribosomal proteins (MRPS30, MPRS15, MPRS9), were upregulated. The upregulation of these mitochondrial enzymes and ribosomal proteins may be a compensatory response to counteract the deterioration of mitochondrial energy metabolism. Five more proteins were also upregulated and involved in mitochondria structural assembly and molecule trafficking (TSPO, PARL, TMEM126A, RHOT2, TOMM40L). In contrast, the only two downregulated proteins consisted of one translation initiation factor (EIF5B) and one sequestosome protein involved in mitochondria dynamics (SQSTM1). Two additional categories involving a high percentage of mitochondria-localized proteins were oxidative stress and lipid metabolism, both of which have been reported to play a role in AMD pathology. Among the seven proteins involved in oxidative stress, four were upregulated, including two glutathione peroxidases (GPX3, GPX4) and two other enzymes localized in mitochondria (PDK1, ADRPS); three were downregulated, including two double-stranded RNA-binding protein Staufen homologs (STAU1, STAU2) and one apoptosis-related kinase (RIPK1). Conversely, among the eleven proteins involved in lipid metabolism, seven were upregulated, including two mitochondrial proteins (TSPO, GPX4), two non-mitochondrial lipases (LIPL, LIPE), and three additional mitochondria-ER-localized enzymes (HDHD5, LPCAT1, PTPMT1); four were downregulated including three members of the Orm family proteins (ORMDL1, ORMDL2, ORMDL3), which negatively regulate sphingolipid synthesis and one phosphatidate phosphatase LPIN1. Taken together, these results provide an in-depth view of novel molecular details of the protein changes occurring at the very earliest stage of AMD.

## 3. Discussion

### 3.1. Establishment of a Reliable Workflow for Human RPE Sample Proteomics Analysis

In this study, we performed a quantitative proteomics experiment to investigate global protein content in the RPE organelle-enriched fractions from human AMD donors and healthy age-matched controls. To achieve high-quality quantification of the RPE proteome, we adopted UHR-IonStar for sample preparation and LC-MS data acquisition/processing and integrated several approaches specifically devised for this study. As a result, ~6000 unique proteins were quantified in the 77 samples with excellent reproducibility (~10% CV for >75% of all proteins quantified), among which 881 were assigned to be localized in the mitochondrion, an organelle with detrimental changes previously associated with AMD [7,8,9]. These results were ascribed to not only the introduction of state-of-the-art LC-MS instruments and techniques but also the utilization of an analytical pipeline well optimized for large sample cohorts. Specifically, the SEPOD protocol enabled exhaustive and efficient protein solubilization/digestion from the isolated organelles-enriched fractions, and the use of a pooled protein digest as the QC sample enabled assessment of LC-MS reproducibility.

Blood contamination of the samples procured from human donors could compromise the quality of proteomics data in several ways. (i) MS detection of peptides from the target proteins could be interfered with by the high-abundance, co-eluting peptides from blood proteins, resulting in a sharp decrease of identifiable peptide/protein number; (ii) the presence of blood proteins can shift the distribution of protein MS intensities, thus causing quantitative biases. Complete blood removal by whole-body perfusion, which is frequently implemented before sample procurement from animal subjects [18], was not possible for clinical samples. Typically, sample qualification is limited to manual inspection, although this method is prone to subjective biases. Hence, a straightforward and robust approach for sample qualification is in urgent demand.

In recent years, several studies have evaluated the impacts of blood contamination on LC-MS-based proteomics data quality in both biofluid and tissue samples. For example, You et al. investigated the proteome of cerebrospinal fluid (CSF) spiked with whole blood from low to high amounts and identified four blood contamination markers (hemoglobin, catalase, peroxiredoxin, and carbonic anhydrase I) for CSF proteomics analysis [19]. In another study, Geyer et al. performed a comprehensive analysis to assess the impacts of erythrocyte and platelet contamination in plasma proteomics via analyzing plasma samples spiked with different amounts of erythrocyte/platelet protein lysates [17]. A list of quality markers for evaluating erythrocyte and platelet issues was provided as a guideline for plasma sample qualification. In the current study, we utilized this information and compiled a list of the most abundant erythrocyte and platelet quality markers plus ALBU that were detected in the current dataset as the serum/plasma marker. Using 10% total MS intensities as the cutoff, we identified six samples containing significant blood contaminants and removed these six samples from the dataset (plus one sample with abnormal MS intensities). Elimination of these “outliers” significantly diminished the intra-group variation of proteomics data, especially the early AMD group (~10% decrease). In addition, we also found that besides using all 16 proteins as quality markers, using the three hemoglobin proteins (HBA, HBB, and HBD) plus ALBU with a 5% cutoff threshold also yielded the same set of outlier samples and, therefore, could also be considered an alternative for the simplicity reason. This compilation of blood contaminant markers as a quality control measure is extremely valuable and applicable for our future proteomics investigation of human RPE samples.

### 3.2. Exploring the Molecular Basis of RPE Mitochondria Dysfunction in Early AMD

RPE has been regarded as the primary site of pathological changes during AMD onset. While the mechanism responsible remains unresolved, our laboratory and others have suggested that mitochondrial dysfunction is a key event in AMD pathology. Furthermore, we have shown mitochondrial DNA damage occurs in the RPE of AMD donors but not in the neural retina, indicating the pathogenic mechanism may be cell-specific [20].

In our previous studies, we investigated RPE proteomic changes at different stages of AMD using 2-DE coupled to either MALDI-TOF or LC-MS/MS [9,21,22]. In the first study, we identified eight spots with significant changes in AMD samples, including three ATP synthase subunits, cytochrome c oxidase VIb, mitofilin, mtHsp70 and mitochondrial translation factor Tu, which are proteins that reside in the mitochondria [21]. Our recently published study identified 58 proteins with altered expression when comparing normal aging with AMD. Many of these altered proteins, with the majority localized to the mitochondria, were associated with both normal aging as well as during AMD progression. However, the direction of change was predominantly opposite, suggesting that aging and AMD involve different biological processes [22].

In the current study, we utilized UHR-IonStar, which allows a much deeper proteome profiling and better quantification, to identify altered proteins (APs) from organelle-enriched fractions in age-matched donors without AMD (MGS1) versus with early AMD (MGS2). Fractionation of RPE allowed us to obtain a more in-depth analysis of proteins associated with cytosolic organelles, including mitochondria. The comparison of proteomic profiles between MGS1 and MGS2 provided valuable information about early proteomic changes in the transition between healthy aging and AMD onset. How these changes set into motion the pathology associated with AMD is still unclear, as our report from human donor tissue cannot discriminate whether the changes are a consequence of cellular stress or whether they are the cause of the pathology observed in the later stages of the disease. Nonetheless, this study adds important new information about potential causative mechanisms associated with the described clinical and biochemical phenotypes of AMD, which could be further tested using experimental approaches that can be genetically or chemically manipulated. Additionally, supervised machine learning analysis identified 19 proteins enabling the classification of the majority of MGS2 and MGS1 samples. While we acknowledge that investigations done in the current study were far from enough for biomarker discovery, the proteins selected by both machine learning algorithms represent a panel of proteins of high biological importance in AMD onset and might contribute to the development of biomarkers for AMD early diagnosis.

Among the 132 APs identified in this study, 39 APs were localized at mitochondria and were involved in the majority of biological processes identified from the AP functional analysis (17 out of 20) and in the PPI networks (Figure 6), including mitochondrial translation and organization, sphingolipid and lipid metabolism, aerobic respiration and oxidative stress. These results indicate significant changes in the mitochondrial proteome in early AMD could have a major impact on critical cellular signaling pathways/biological processes.

Additionally, the majority of mitochondrial-related APs were upregulated in MGS2 samples, indicating a potential compensatory response in RPE cells during early AMD.

For example, the upregulation of translational proteins in the MGS2 RPE suggested proteome alterations, which may include both pathological changes as well as adaptive changes in the RPE of early-stage AMD. Three mitochondrial ribosomal proteins (MRPS30, MPRS15, MPRS9) and five proteins involved in mitochondria trafficking and assembly (TSPO, PARL, TMEM126A, RHOT2, TOMM40L) were increased, indicating a potential compensatory response to mitochondrial damage caused by disease conditions. The upregulation of four enzymes of glycolysis (HK1, HK2, PKM, OGDHL) in MGS2 samples is consistent with the increased glycolytic proteins in RPE with AMD [22]. Interestingly, some enzymes involved in oxidative phosphorylation were also increased at the early stage. It can be speculated that in the transition from normal to early AMD status, the RPE counteracts energy metabolism defects by elevating both mitochondrial and glycolytic protein expression levels in an effort to meet cellular energy demands.

Another class of proteins significantly changed in MGS2 is involved in oxidative stress. Oxidative stress has been regarded as a significant contributor to AMD pathophysiology [23]. Abundant mitochondria present in RPE are required for maintaining the highly active metabolism in RPE cells. ROS, generated as the consequence of mitochondrial metabolism, can contribute to the oxidative burden in RPE. Smoking or a high fat diet, also significant risk factors for AMD, could impose extra oxidative stress on RPE cells. In this study, our data suggest that oxidative stress was present in the RPE experiencing early AMD. For example, the upregulation of PDK1 (pyruvate dehydrogenase kinase 1) in MGS2 samples indicated the inactivation of pyruvate dehydrogenase and, thus, disrupting the homeostasis of carbohydrate fuels. ADPRS (ADP-ribosylserine hydrolase) plays a key role in DNA damage response; GPX3 and GPX4 (glutathione peroxidases) protect cells against oxidative damage. The elevation of these proteins and the downregulation of apoptosis-related kinase (RIPK1) in MGS2 samples suggested a protective mechanism against oxidative damage during AMD onset. Of note, the double-stranded RNA-binding protein Staufen homologs (STAU1, STAU2), two essential components of stress granules, were decreased in the MSG2 samples. It has been shown that STAU1 is involved in recovery from stress by stabilizing polysomes and helping stress granule dissolution [24]. This data suggests the recovery from oxidative stress may be compromised in the RPE of donors with early AMD.

Dysregulated lipid or lipoproteins has long been regarded as a significant phenotype of AMD since lipoproteins and cholesterol are major constituents of drusen. Sphingolipids are bioactive molecules associated with oxidative stress and inflammation. Increasing evidence supports that altered sphingolipid levels contribute to AMD pathology [25]. Ceramide has also been shown to be a crucial player in the induction of RPE cell death. In this study, we found a decrease in ORMDL proteins, which are negative regulators of sphingolipid biosynthesis, indicating a potential increase of sphingolipids in RPE cells in the early stage of AMD. We also found upregulation of LPL, LIPG (both are involved in triglyceride metabolism), PTPMT1, and HDHD5 (both are involved in glycerophospholipid biosynthesis and metabolism) in MGS2 samples. LPIN1 regulates general lipid metabolism by entering the nucleus, where it inhibits SREBP activity and reduces the expression of lipid genes [26]. The downregulation of LPIN1 suggested a potential upregulation of lipid biosynthesis in the RPE of early AMD.

Since no animal model fully replicates the cardinal features of AMD, the use of human donor tissue provides the best way to investigate AMD mechanism. However, there are several caveats associated with our experimental design that need to be recognized. While we were able to match ages and gender distribution in our two groups, we had no control over other factors, such as diet and lifestyle, that could influence our results. Another consideration is that our preparation of organelle-enriched fractions would miss protein changes in other cellular locations. Therefore, our results likely underestimate the total changes present in early AMD. Finally, while we cannot infer causality as protein changes simply correlate with disease, these results provide a roadmap for future investigations into disease mechanisms and the discovery of targets for therapy.

## 4. Materials and Methods

### 4.1. Human Eye Procurement and Grading for AMD

De-identified donor eyes were obtained from the Lions Gift of Sight (Saint Paul, MN, USA). Eyes were obtained with the written consent of the donor or donor’s family for use in medical research in accordance with the Declaration of Helsinki. The Lions Gift of Sight is licensed by the Eye Bank Association of America (accreditation #0015204) and accredited by the FDA (FDA Established Identifier 3000718538). De-identified donor tissue is exempt from the process of Institutional Review Board Approval.

Tissue handling, storage and donor exclusion criteria are as outlined previously [27]. Evaluation of the presence or absence of AMD was determined by a Board-Certified Ophthalmologist (Dr. Sandra R. Montezuma) from stereoscopic fundus photographs of the RPE using a set of criteria (RPE pigment changes and presence, drusen size and location) established by the Minnesota Grading System (MGS) [11].

### 4.2. Sample Preparation for Proteomics Analysis

RPE cell pellets from individual donors were suspended in an isolation buffer (70 mM sucrose, 200 mM mannitol, 1 mM EGTA, 10 mM HEPES, pH 7.4) and subjected to two freeze-thaw cycles prior to homogenization. Samples were centrifuged at 800× *g* for 8 min, and the supernatant (containing the organelles) was centrifuged at 12,000× *g* for 10 min. The pelleted organelle-enriched fraction was resuspended in an ice-cold SC buffer (50 mM Tris-formic acid, 150 mM NaCl, 2% SDS, 0.5% sodium deoxycholate, 2% IGEPAL CA630, pH 8.4) supplemented with complete protease inhibitor cocktail tablets (Roche Applied Science, Indianapolis, IN, USA), and sonicated in a water bath sonicator for 10 min to solubilize proteins. Protein concentration was determined by bicinchoninic acid assay.

For protein digestion, 60 μg protein was aliquoted from each sample, and an SC-aided precipitation/on-pellet digestion (SEPOD) protocol was employed as previously described [14]. Protein was first reduced by 10 mM dithiothreitol (DTT) at 56 °C for 30 min and alkylated by 25 mM iodoacetamide (IAM) at 37 °C for 30 min in darkness. Both steps were performed with constant shaking (550 rpm) in a covered thermomixer (Eppendorf, Framingham, MA, USA). Protein precipitation was performed by the addition of 6 volumes of ice-cold acetone with vigorous vortexing, and the mixture was incubated at −20 °C for 3 h. Precipitated protein was pelleted by centrifugation at 18,000× *g*, 4 °C for 30 min, and was gently washed with 500 μL methanol. After removing the supernatant, the protein pellet was left to air dry for 1 min, and 48 μL 50 mM Tris-formic acid (FA), pH 8.4 was added to wet the pellet. A total volume of 12 μL trypsin (Sigma-Aldrich, St. Louis, MO, USA) dissolved in 50 mM Tris-FA (0.25 μg/μL) was added to each sample to reach a final enzyme-to-substrate ratio of 1:20 (*w*/*w*), and samples were incubated in a covered thermomixer at 37 °C for 6 h with constant shaking. Tryptic digestion was terminated by the addition of 0.6 μL FA, and samples were centrifuged at 18,000× *g*, 4 °C for 30 min. The supernatant was carefully transferred to LC vials for analysis.

### 4.3. Liquid Chromatography-Mass Spectrometry (LC-MS)

The LC-MS system consists of a Dionex UltiMate 3000 nano-LC system, a Dionex UltiMate 3000 micro LC system with a WPS-3000 autosampler, and an Orbitrap Fusion Lumos mass spectrometer (ThermoFisher Scientific, San Jose, CA, USA). A large-inner diameter (i.d.) trapping column (300-μm i.d. × 5 mm; Agilent Technologies, Santa Clara, CA, USA) setting was implemented before the analytical column (75-μm i.d. × 65 cm, packed with 2.5-μm XSelect CSH C18 material) for high-capacity sample loading, cleanup and delivery. For each sample, a peptide equivalent to 4 μg protein was injected for LC-MS analysis. Mobile phases A and B were 0.1% FA in 2% acetonitrile (ACN) and 0.1% FA in 88% ACN. The 180-min LC gradient profile for peptide separation was: 4% B for 3 min, 4–11% B for 5 min, 11–32% B for 117 min, 32–50% B for 10 min, 50–97% B for 1 min, 97% B for 17 min, and then equilibrated to 4% for 27 min. The mass spectrometer was operated under data-dependent acquisition (DDA) mode with a maximal duty cycle of 3 s. MS1 spectra were acquired by Orbitrap (OT) under 240k resolution for ions in the *m*/*z* range of 400–1500. Automatic Gain Control (AGC) target and maximal injection time were set to 175% and 50 ms, and dynamic exclusion was set as 60 s, ±10 ppm. Precursor ions were isolated by quadrupole using an *m*/*z* window of 1.6 Th and were fragmented by high-energy collisional dissociation (HCD) at 30% energy. MS2 spectra were acquired by OT under 15k resolution with an AGC target of 100% and a maximal injection time of 50 ms. Detailed LC-MS set-tings and information can be found in our previous publications [12,13,15].

### 4.4. LC-MS Data Processing

An in-house developed UHR-IonStar data processing pipeline was adopted for proteomic quantification. Database searching was conducted by matching the LC-MS raw files against the human Swiss-Prot protein sequence database (20,302 entries, downloaded in May 2020) using the MS-GF+ search engine (v20210108, released in January 2021). Search parameters included: (1) Precursor mass tolerance: 20 ppm; (2) Instrument type: Q-Exactive; (3) Matches per spectrum: 1; (4) Fixed modification: carbamidomethylation of cysteine (C); (5) Dynamic modification: oxidation of methionine (M) and acetylation of peptide N-terminal; (6) Maximal missed cleavages: 2. Peptide-spectrum match (PSM) filtering, protein inference and grouping, and global false discovery rate (FDR) control were accomplished by IDPicker (v3.1.18192.0). Protein and peptide FDR were controlled at ≤1%, and a minimum of 2 unique peptides per protein was set. Proteins with no unique peptides were grouped with a maximal number of 50 proteins per group. The filtered PSM list was generated by the UHR-IonStar APP (https://github.com/JunQu-Lab/UHRIonStarApp, accessed on 29 March 2022) using lists of proteins/peptides/spectra exported from IDPicker.

Peptide quantitative features were extracted from LC-MS raw files using SIEVE (v2.2, ThermoFisher Scientific, San Jose, CA, USA) and further processed by the UHR-IonStar APP to generate protein quantification results. Primary procedures included: (1) chromatographic alignment with ChromAlign [28] for dataset-wide retention time (RT) adjustment and peak clustering. The optimal reference run was selected based on alignment scores in the entire dataset; (2) data-independent MS1 quantitative feature extraction using the direct ion-current extraction (DICE) method, which uses a pre-defined *m*/*z*-RT window (10 ppm, 1 min for 240k MS1) to extract ion chromatograms for all precursor ions subjected to fragmentation and MS2 acquisition in the aligned dataset. Each set of extracted ion chromatograms with corresponding area under the curve (AUC) in all files is termed as one “frame”; (3) integration of the SIEVE frame database and the filtered PSM list by a unique identifier combining file name and MS2 scan number. Frames with valid peptide sequences were then subjected to frame-level quality control, global normalization, peptide-level outlier detection/exclusion, and aggregation to the protein level. More detailed information about the UHR-IonStar data processing pipeline can be found in our previous publications [12,13].

### 4.5. Data Analysis and Bioinformatics

Quantification results were further processed using the UHR-IonStar APP, which encompassed the following steps: (1) data formatting and cleanup; (2) statistical testing by Welch’s *t*-test; (3) calculation of protein ratios between MGS2 and MGS1 groups. Gene Ontology (GO) enrichment of Cellular Component terms was performed by the Database for Annotation, Visualization, and Integrated Discovery (DAVID) Bioinformatics Resources (https://david.ncifcrf.gov/, accessed on 10 April 2022) [29,30], and results were manually curated. Machine learning analysis of APs was performed with random forest (RF) and support vector machines (SVM) using *caret* package in R [31]. Model accuracy was estimated by tenfold cross-validation, and the procedure was repeated three times. Tuning parameters were determined when the model hit the best accuracy. The tuning parameters used were: (1) RF: ntree =1000 (number of trees to grow); mtry = 106 (number of variables randomly sampled as candidates at each split); (2) SVM: cost = 1 (cost of constraints violation and the regularization term in the Lagrange formulation). Feature importance for RF and SVM were calculated by “mean decrease in accuracy” and ROC curve analysis, respectively. Annotation of protein functions and pathways, as well as protein–protein interaction analysis, were performed by Metascape (https://metascape.org/, accessed on 27 June 2022) [32] and visualized using Cytoscape v3.9.1 [33]. Hierarchical clustering, Principal Component Analysis (PCA) and Partial Least Squares-Discriminant Analysis (PLS-DA) was performed by R using corresponding packages. All other data visualization was done by Graphpad Prism and R with the ggplot2 package.

## 5. Conclusions

In this study, we employed UHR-IonStar to investigate the proteomic profiles of RPE from donors with early AMD compared with healthy age-matched controls. Notably, a standardized scheme for monitoring LC-MS reproducibility and an outlier sample detection procedure was implemented to achieve high-quality quantification of the RPE proteome. Our data suggest that during the transition to early AMD, the RPE alters metabolism, exhibits an oxidative stress response, and upregulates lipid biosynthesis. These adaptive mechanisms are likely to counteract detrimental cellular conditions by elevating mitochondrial translational/trafficking/assembly proteins and increasing energy metabolism.

## Data Availability

The LC-MS data have been deposited to the ProteomeXchange Consortium via the PRIDE [34] partner repository with the dataset identifier PXD033413 and DOI: 10.3390/CELLS11132075. UHR-IonStar and the user manual are available online (https://github.com/JunQu-Lab/UHR-IonStar, accessed on 1 November 2022).

## Supplementary Materials

The following supporting information can be downloaded at: https://www.mdpi.com/article/10.3390/ijms24043252/s1.

## Abbreviations

2-D	Two-dimensional
AGC	Automatic Gain Control
ALBU	Albumin
AMD	Age-related macular degeneration
AP	Altered protein
BP	Biological process
CC	Cellular Component
CSF	Cerebrospinal fluid
CV	Coefficient of variance
CFH	Complement factor H
DAVID	Database for Annotation, Visualization, and Integrated Discovery
DIA	Data-independent acquisition
DICE	Direct Ion-Current Extraction
DDA	Data-dependent acquisition
DTT	Dithiothreitol
FA	Formic acid
FDR	False discovery rate
GO	Gene Ontology
HCD	High-energy collisional dissociation
IAM	Iodoacetamide
LC	Liquid chromatography
MALDI	Matrix-assisted laser desorption ionization
MGS	Minnesota Grading System
MS	Mass spectrometry
OT	Orbitrap
PSM	Peptide-spectrum match
PCA	Principal Component Analysis
PLS-DA	Partial Least Squares-Discriminant Analysis
PPI	Protein-protein interaction
QC	Quality Control
RF	Random Forest
RDH5	retinol dehydrogenase
RGR	retinal G protein-coupled receptor
RPE	Retinal pigment epithelium
RPE65	retinoid isomerohydrolase
SC	Surfactant cocktail
SEPOD	Surfactant cocktail-aided extraction/precipitation/on pellet digestion
SNP	Single nucleotide polymorphism
SVM	Support Vector Machine
TOF	Time-of-flight
UHR	Ultra-High-Resolution
