# Supplementary material for: Quantitative Proteomics of Human Retinal Pigment Epithelium Reveals Key Regulators for the Pathogenesis of Age-Related Macular Degeneration
Source: Int J Mol Sci. 2023 Feb 7;24(4):3252. doi: 10.3390/ijms24043252 (PMC9959910; doi:10.3390/ijms24043252)
Supplement: Supplementary file 1 [file ijms-24-03252-s001.zip › Supplementary materials.pdf]

Large-Scale Quantitative Proteomics of Human Retinal Mitochondria-Enriched Fractions Reveals Key Regulators for the Early Pathogenesis of Age-Related Macular Degeneration

Shichen Shen [1,2], Rebecca J. Kapphahn [3], Ming Zhang [1,2], Shuo Qian [4], Sandra R. Montezuma [3], Peng Shang[5], Deborah A. Ferrington [3,5,*], and Jun Qu [1,2,*]

[1] Department of Pharmaceutical Sciences, University at Buffalo, Buffalo, NY 14214, USA
[2] New York State Center of Excellence in Bioinformatics and Life Sciences, Buffalo, NY 14203, USA
[3] Department of Ophthalmology and Visual Neurosciences, University of Minnesota, Minneapolis, MN 55455, USA
[4] Department of Cell Stress Biology, Roswell Park Comprehensive Cancer Center, Buffalo, NY 14203, USA
[5] Doheny Eye Institute, Pasadena, CA 91103
* Correspondence: DFerrington@doheny.org (D.A.F.); JunQu@buffalo.edu (J.Q.); Tel.: +1-(323)342-6404 (D.A.F.); +1-(716)645-4821 (J.Q.)

Supplementary Materials

Supplementary Figure S1 – S6

Supplementary Table S1 – S7

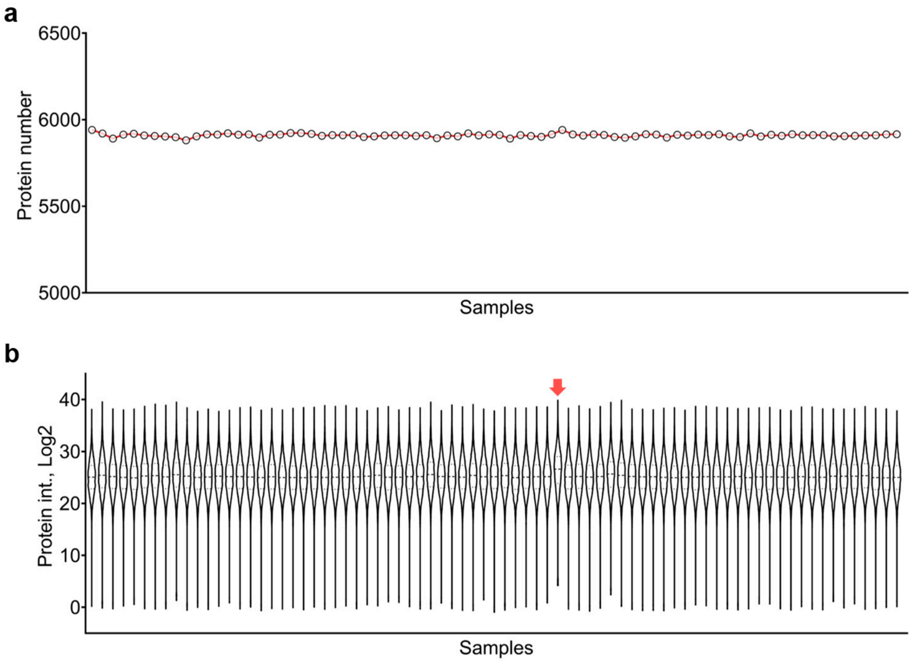

Supplementary Figure S1. Proteomic profiles of the 77 retinal pigment epithelium (RPE) mitochondria-enriched fraction samples. (a) Number of proteins quantified in individual samples; (b) Log2 protein mass spectrometry (MS) intensities of individual samples. The red arrow denotes the 1 sample (M2_S2_2) with abnormal MS intensities.

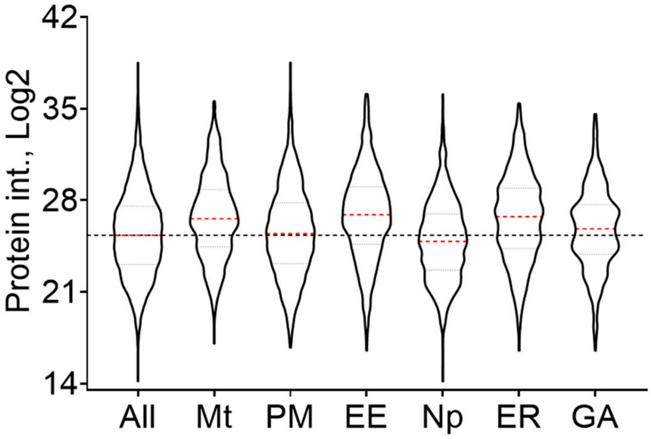

Supplementary Figure S2. MS intensities distribution of proteins enriched in major cellular compartments. All: all proteins; Mt: mitochondrion; PM: plasma membrane; EE: extracellular exosomes; Np: nucleoplasm; ER: endoplasmic reticulum; GA: Golgi apparatus.

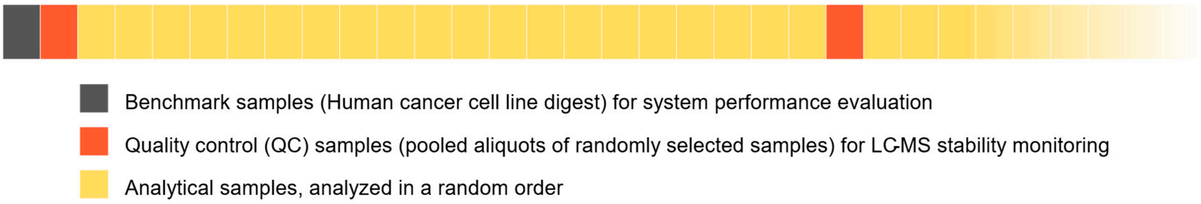

Supplementary Figure S3. A standardized LC-MS scheme allowing monitoring and evaluation of reproducibility during the analysis queue.

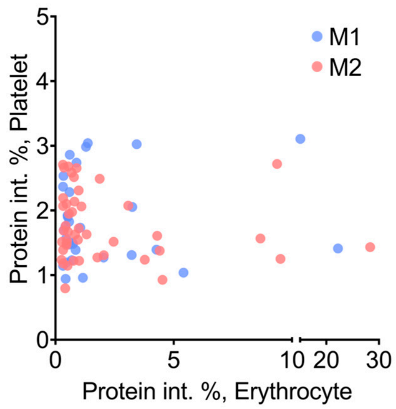

Supplementary Figure S4. Correlation plot between the percentage of erythrocyte- and platelet-specific proteins in all samples.

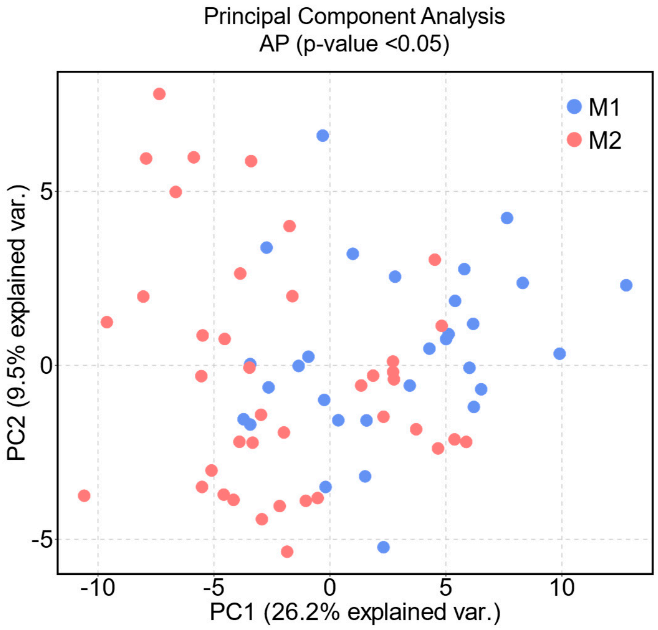

Supplementary Figure S5. Principal Component Analysis (PCA) of M1 and M2 samples using the 132 altered proteins (APs).

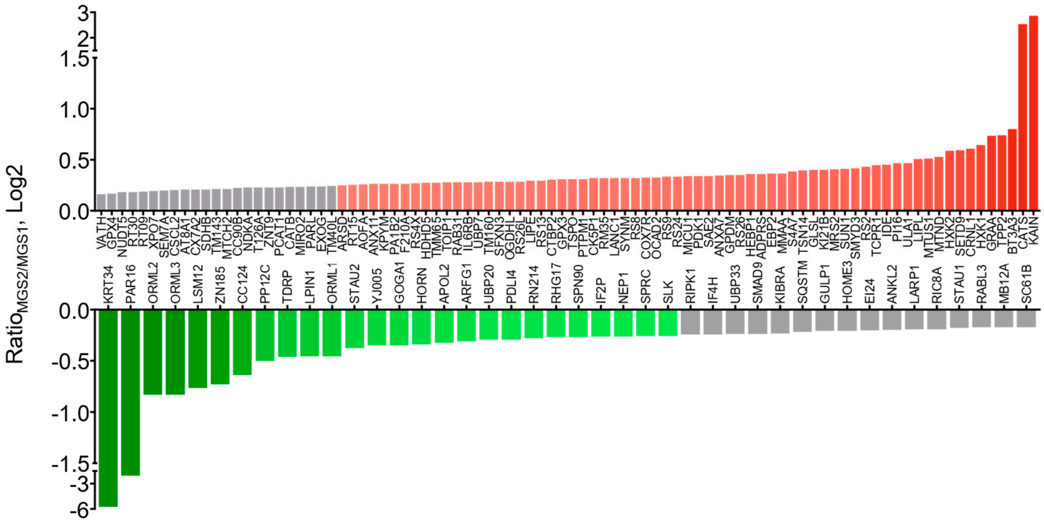

Supplementary Figure S6. APs ranked by the magnitude of fold changes. Red and green bars denote APs with Log_2 ratio >0.25 or <-0.25 (approximately ~20% fold change).

Supplementary Table S1. Demographic profiles of donors involved in the current study.

Supplementary Table S2. Quantification results of 5,941 proteins in 77 RPE mitochondria-enriched fractions from human donors w/ and w/o AMD.

Supplementary Table S3. Proteomic quantification results of the 5 QC sample runs.

Supplementary Table S4. Percentage of MS intensities of the 16 quality markers within each sample. Samples removed are highlighted in red.

Supplementary Table S5. Quantification results of 5,941 proteins after removing 7 outlier samples with high blood protein percentages and abnormal MS intensities.

Supplementary Table S6. A total of 132 altered proteins (APs) determined by Welch's t-test.

**All Supplementary Tables are provided as separate Excel files.
